# Caregiver and clinician perceptions of barriers to cerebral palsy healthcare—mixed methods findings and systems change recommendations

**DOI:** 10.3389/fpubh.2025.1644144

**Published:** 2025-10-22

**Authors:** Melissa M. Murphy, Gavin Colquitt, Paige S. Ryals, Katie Shin, William C. Kjeldsen, Nathalie L. Maitre

**Affiliations:** ^1^BBOP Lab, Division of Neonatology, Emory University School of Medicine, Atlanta, GA, United States; ^2^Appalachian Institute for Health and Wellness, Appalachian State University, Boone, NC, United States; ^3^Children’s Healthcare of Atlanta, Atlanta, GA, United States

**Keywords:** cerebral palsy, needs assessment, clinician perspectives, trainee pathways, clinical access, disability community engagement

## Abstract

**Introduction:**

The present paper is the second in a series of *exploration phase* efforts toward building and sustaining patient-centered agendas for research and clinical care in CP.

**Methods:**

Focus group and surveys were used to assess perspectives of caregivers of young children with CP (*N* = 19) and clinicians (*N* = 102) regarding CP-specific medical care priorities and barriers and facilitators to high quality CP-focused care in Georgia, US.

**Results:**

Qualitative and quantitative analysis reveal areas of areas of synergy and discrepancy between the two stakeholder groups.

**Discussion:**

Together stakeholder responses converge on the notion that (1) *empowering caregivers* to better utilize the resources that do exist and (2) *building provider capacity and confidence* in efficient delivery of high-quality CP care is critical to drive system changes for improving access and outcomes across the lifespan. Proposed action items for systems change arise from the convergence of caregiver and clinician responses.

## 1 Introduction

Cerebral palsy (CP) is the most common lifetime physical disability affecting more than 40,000 people in Georgia (1). Per person with CP, lifespan costs to families exceed $1.3 million, adjusted for inflation (2). CP manifests as a spectrum of phenotypes resulting from variable, non-progressive insults during the perinatal period (3). The condition leads to movement disorders and secondary conditions that severely impact quality of life and independence (4). Risks are heightened by factors including maternal health, perinatal factors, pregnancy complications, and healthcare disparities (5). The complexity of care needs in this population requires patient-provider shared decision making (6, 7). High-quality care in early CP involves multiple components as proposed in various care guidelines: use of evidence-based care and practices in an interdisciplinary, lifespan- and patient/family-centered approach, from early detection and diagnosis to surveillance of co-occurring conditions and connection of families with appropriate intervention services, addressing socio-economic resource issues and psychological concerns for families and patients with CP (8–11). Yet, barriers to clinical care for both patients and providers can impede efforts to provide high-quality patient care in CP. These effects may be especially pronounced in the early years, where diagnostic conversations and interactions with clinical services can shape how caregivers experience a diagnosis of CP (12, 13) and the subsequent impact of information perception over time (12, 14).

**Figure 1:**
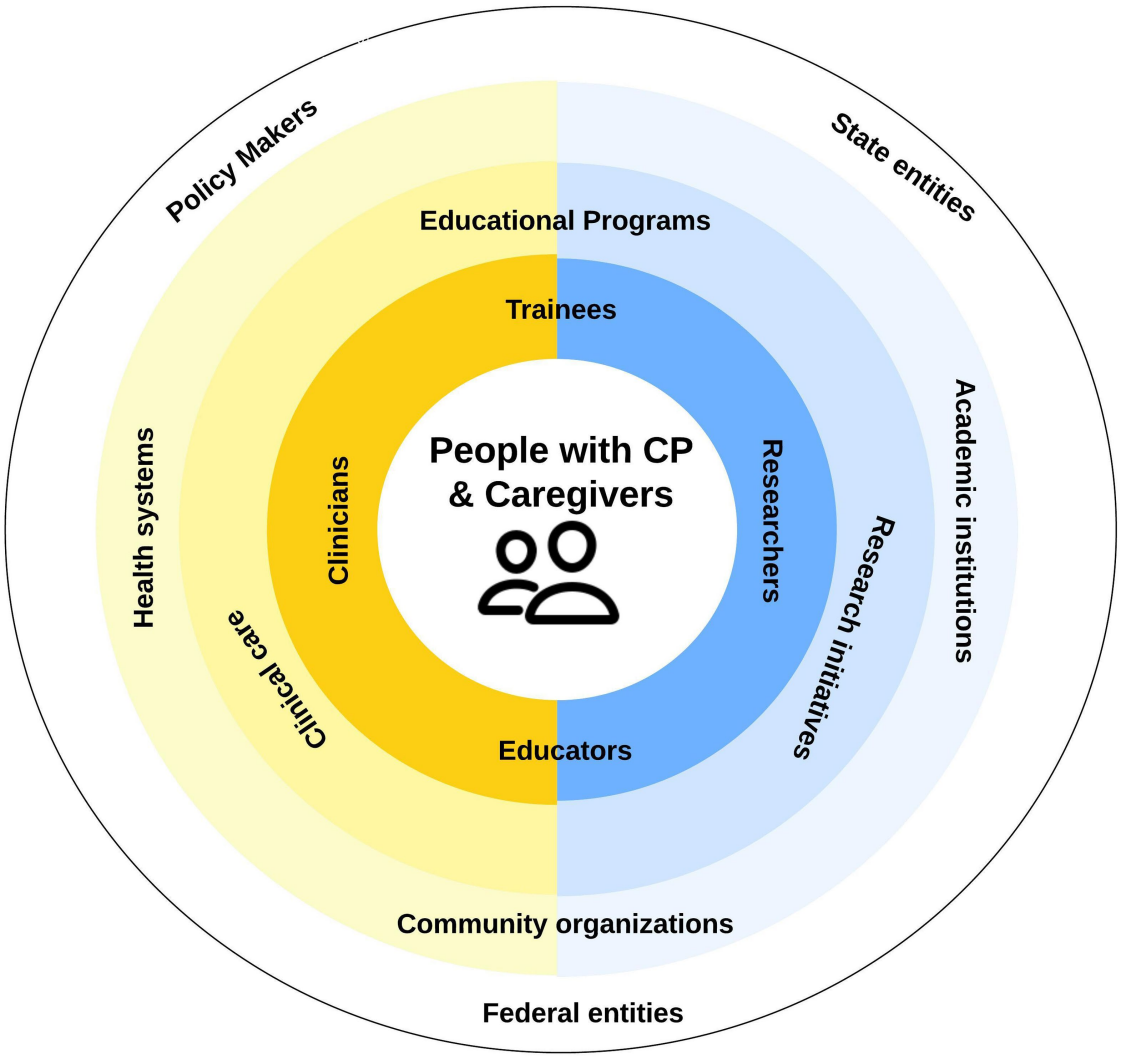
Systems view of ideal state of cerebral palsy (CP) clinical research efforts. Barriers exist at all levels impeding progress toward high-quality CP care. Adapted from Murphy et al. (17).

The International Classification of Functioning, Disability, and Health (ICF) framework, commonly applied in CP (15, 16), emphasizes the interaction of physical, mental and socioeconomic factors in health outcomes of children with CP (15). This framework is however complemented by a bio-ecological systems perspective that considers the ways in which multiple levels and influences on healthcare systems interact to influence development across the lifespan. From these perspectives, improving health outcomes for people with CP means identifying and addressing barriers—and strengthening facilitators—to high-quality clinical care related to biological, psychological, and social factors experienced by the individual and at every level of the healthcare system [Figure 1; (17)]. Given the system complexity, shared decision-making approaches to managing complex healthcare needs are increasingly being adopted to improve outcomes (6, 7). Further, efforts to encourage clinicians and lived experience partners (e.g., people with CP and parents of a child with CP) to “codesign” pathways addressing barriers to high-quality care are underway (17–19).

Murphy et al. (17) conducted a statewide engagement study to understand synergies and discrepancies in stakeholder perspectives can help to support aligning priorities, promoting connection, and nurturing trust across all levels of the system in clinical research (17). In this study and others (20), participatory action research (PAR) approaches to research have been successfully used in CP research to generate action steps and accountability (17, 20).

The current study sought to employ a PAR approaches to build and sustain patient-centered agendas, this time focused on clinical care in CP. Caregivers of a young child with CP and medical or allied health clinicians (e.g., therapist, social workers, kinesiologists) provided their perspectives on medical care priorities, and barriers and facilitators to high quality CP-focused medical care in Georgia, US. By focusing on a state in the US with frequent perinatal risk factors for CP mortality (21), we explored region-specific factors affecting CP care leading to targeted action items with potential implications for similar US states (17).

## 2 Methods

### 2.1 Design

We employed a convergent parallel mixed-methods design that uses side-by-side comparison to triangulate stakeholder perspectives on high-quality CP care (22). Focus groups and quantitative surveys were used to document the current state of CP clinical care and identify key actions to guide system-level improvements in clinical care and health outcomes for people with CP.

### 2.2 Ethics statement

This quality improvement initiative was designated as not “human subjects research” by the Emory Institutional Review Board. Surveys were anonymous. Graphical recordings of caregiver responses did not link identifiers and responses.

### 2.3 Setting

Perspectives of clinicians and caregivers were collected during the *2023 Cerebral Palsy Foundation Early CP Health Summit*, an international, interdisciplinary implementation-focused meeting for medical and allied health professionals translating CP early detection and intervention knowledge into practice for the birth to 3 year age range. A track for caregivers of a young child with CP was added at the 2023 Summit. To ensure a more complete representation of Georgia persepectives, clinicians in Georgia’s perinatal regionalization high risk infant follow-up (HRIF) clinics were also invited to complete the survey during an intensive training on early detection led by the senior author (NLM).

### 2.4 Participants

All caregivers and CP early detection initiative (EDI) clinician attendees at the Early CP Health Summit and the HRIF clinic trainings were invited to participate. Demographics for the full sample are published elsewhere (17). Table 1 presents select characteristics by partner group (caregiver *N* = 19, clinician *N* = 102).

**Table 1:** Participant demographic characteristics by group: Caregivers, Clincians.

Characteristics by group	Number (%)
Caregivers (*n* = 19)	
Age (% ≥ 30 years)	18 (79%)
Relationship to child with cerebral palsy (% Mother)	14 (74%)
Education level (% partial college or more)	15 (80%)
Child characteristics	
Age (% 1–3 years)	15 (79%)
Clinicians (*n* = 102)	
Profession	
Therapist (Music, Occupational, Physical, Speech Language)	66 (65%)
Medical Doctor	22 (22%)
Nurse/Nurse Practitioner	10 (9%)
Other	4 (4%)
Clinician Recent/Primary Position (% Academic Hospital/System)	50 (49%)
Position Geographical Region (% Eastern US)	62 (61%)
Length in Position (% ≥ 10 years)	52 (50%)
Special Access Requirements/ADA Accommodations (% Yes)	1 (1%)

Note: Table is modified version of full table presented in Murphy et al. (17).

### 2.5 Data collection

Guided by a convergent parallel mixed-methods design, we captured both numerical ratings and narrative insights during the 2023 Early CP Health Summit and the HRIF trainings. A brief overview of these complementary procedures is provided here, with complete methodological details reported elsewhere (17).

#### 2.5.1 Quantitative

A 0–100 sliding scale, chosen because the continuous slider provided finer resolution than a 5- or 7-point Likert scale, captured subtle distinctions in perceived importance and implementation barriers, and mitigated central-tendency and ceiling effects. Using the sliding scale from 0 (not at all) to 100 (very much so), clinicians rated the importance of specific clinical objectives to creating and maintaining a high-quality CP clinical program. These objectives (see Table 2) were based upon an educational bundle developed for the CPF Early Detection of CP Network and existing guidelines for CP care (8–11, 23). They also rated barriers and facilitators to implementing these clinical objectives using the same sliding scale. Caregivers used the same scale to rate the importance of options for their child’s medical care and degree of difficulty accessing medical care. They were also asked to rate their preference for community opportunities, educational topics, and types of therapy services.

**Table 2:** Clinician ratings of importance, frequency, and barriers of implementation of clinical objectives for creating and maintaining a high-quality clinical program for treating patients with CP (*N* = 102) on scale 1–100 with 100 being very much so: Median (10th, 90th percentile).

Clinical objectives	Median (10th, 90th)
Clinical objectives – importance	
Connecting families with appropriate intervention services (e.g., early intervention, Babies Cannot Wait, PT/OT/SLP referrals, specialist referrals)	100 (82, 100)
Using a patient/family-centered approach to care	100 (82, 100)
Addressing socio-economic resource issues for families and patients with CP	100 (72, 100)
Use of evidence-based care and practices	100 (76, 100)
Addressing psychological concerns for families and patients with CP	100 (73, 100)
Interdisciplinary approach (e.g., medical doctors/nurse practitioners work together with therapists and other specialists to provide coordinated care)	100 (80, 100)
Providing access to support services across the lifespan for people with CP	100 (78, 100)
Early detection and diagnosis for patients with CP	100 (75, 100)
Clinical objectives – frequency of implementation by clinical program	
Connecting families with appropriate intervention services (e.g., early intervention, Babies Cannot Wait, PT/OT/SLP referrals, specialist referrals)	90 (50, 100)
Using a patient/family-centered approach to care	88 (58, 100)
Use of evidence-based care and practices	82 (50, 100)
Interdisciplinary approach (e.g., medical doctors/nurse practitioners work together with therapists and other specialists to provide coordinated care)	80 (50, 100)
Early detection and diagnosis for patients with CP	70 (35, 100)
Addressing socio-economic resource issues for families and patients with CP	62 (31, 94)
Providing access to support services across the lifespan for people with CP	60 (20, 97)
Addressing psychological concerns for families and patients with CP	56 (29, 91)
Clinical objectives – barriers to implementation	
Lack of clinician time	77 (50, 100)
Lack of insurance reimbursement and payment for services	75 (25, 100)
Hard to find appropriate resources for the family	65 (25, 89)
Lack of understanding and opportunities to learn evidence-based research practice	61 (19, 92)
Not a good systematic approach to care in place	61 (15, 100)
Hard to find or access correct specialists	58 (18, 93)
Families not engaged in attending appointments	55 (17, 96)
Hard to find or access most up-to-date research	50 (0, 75)

Note: CP, cerebral palsy; PT, physical therapy; OT, occupational therapy; SLP, speech language therapy.

Both clinician and caregiver surveys were administered via REDCap, a secure, web-based platform for data collection (24, 25). Survey questions and response choices are included in Supplementary Tables S1, S2 for caregivers and clinicians, respectively. Each survey version took 5–10 min to complete.

#### 2.5.2 Qualitative

Caregivers participated in World Café style focus groups on the first afternoon of the Health Summit. After a welcome and introduction, caregivers were prompted to discuss challenges in obtaining high quality health care, therapy, and equipment. Two facilitators from the research team who were not involved in any clinical care were present at each table to assist with graphical recording of responses. Following the small group discussion, co-authors (MMM, PSR) led a whole group discussion to “harvest” (26) responses and increase data validity through real-time member-checking of synthesized data (27, 28). Responses on the graphical recordings were transcribed into Excel and checked for accuracy. Clarifications were noted in brackets within the transcriptions.

## 3 Analysis

Quantitative data were analyzed using SPSS version 29 (29). Descriptive statistics are reported. To account for non-normally distributed data, median ratings are reported along with the 10th and 90th percentile values. Ratings 80–100 were considered “very important,” 60–79 “somewhat important,” 40–59 “important,” 20–39 “somewhat not important,” and 0–29 “not important.” Non-parametric statistics for medians were used for any group comparisons.

Thematic analysis of qualitative data was accomplished using an inductive reasoning approach described in detail elsewhere (17). In short, team members identified initial themes and subthemes within caregiver responses by focusing on the meaning expressed in the statement. Kappa coefficient for interrater reliability for coding was 0.81 (30), indicating strong agreement (31). Any coding disagreements were resolved by discussion among coders. Once caregiver perceptions of challenges to getting care were coded, they were considered alongside clinician ratings of patient barriers to engaging in clinical care with the goal of identifying areas of convergence between the two groups in perceived barriers and facilitators of high-quality CP clinical care.

## 4 Results

### 4.1 Caregiver ratings

Caregiver-rated importance of options for their child’s medical care and barriers to accessing medical care, preferences for community opportunities, and types of therapy services (Table 3) along with preferences for educational opportunities (Table 4). Medians are reported along with inter 10th and 90th percentile range (IPR).

**Table 3:** Caregiver-reported importance of options for child’s medical care and barriers to accessing medical care survey responses (*n* = 19) on scale 1–100 with 100 being very much so: Median (10th, 90th percentile).

Medical care options and barriers to access	Median (10th, 90th)
**With regard to your child’s medical care…**	
How important are the following items to you:	
Options for in-home therapy and/or care services for my child	100 (76, 100)
Easy access to medical providers that know about CP	100 (85, 100)
Easy access to the latest information about interventions and treatments for CP	100 (80, 100)
Understanding my child’s care options	100 (77, 100)
Easy access to the latest information about CP and how it can affect my child’s development	100 (68, 100)
**A care coordinator to help me navigate all these services**	**100 (50, 100)**
**Options for telehealth services for my child**	**71 (0, 100)**
How hard is it for you to access the following items:	
**Medical appointment with primary care**	**27 (0, 100)**
Affordable medications^a^	50 (0, 100)
Occupational or Physical Therapy	50 (8, 100)
Therapeutic equipment (e.g., splits)^b^	50 (6, 100)
Speech or Feeding Therapy	50 (0, 100)
**Medical appointments with specialists (e.g., neurologists, developmental pediatricians)**	**70 (28, 100)**
If the distance or availability of provider were not an issue, and there were no extra costs – how much would you prefer the following types of therapy services:	
In the home – on a weekly basis	95 (50, 100)
In the home – intensive periods for a few weeks followed by rest periods to consolidate skills	75 (27, 100)
In a group setting with families who have children with the same issues	61 (21, 100)
In the clinic – on a weekly basis	71 (0, 100)
In the clinic – intensive periods for a few weeks followed by rest periods to consolidate skills	69 (0, 100)
**Via telehealth – on a weekly basis**	**50 (0, 66)**
Via telehealth – intensive periods for a few weeks followed by rest periods to consolidate skills	50 (0, 92)
**With regard to community opportunities….**	
How important are the following:	
For my family to have opportunities to learn more about the latest updates in caring for a child or person with cerebral palsy	100 (50, 100)
For my child to have opportunities to get to know other children with CP	100 (50, 100)
For my family to have opportunities to participate in studies that help us learn more about CP	94 (50, 100)
For me (the caregiver) to have in-person opportunities to get to know other parents that have children with CP	100 (50, 100)
For me (the caregiver) to have online opportunities to get to talk to other parents that have children with CP	81 (50, 100)

Note: Bold text indicates the prompt stem for each set of options rated; ^a^*n* = 17; 2 = n/a; ^b^*n* = 18; 1 = n/a.

**Table 4:** Caregiver endorsement of educational topics (*n* = 19): number (% endorsing).

Topic	Number (%)
Interventions to improve motor function (e.g., therapy, surgery, medicine)	18 (95%)
Environmental supports to help my child progress	16 (84%)
News updates when something related to my child’s CP happens	16 (84%)
Family and social supports specialized to those with children with CP	16 (84%)
Community/state supports to help my child progress	15 (79%)
Extra resource options that can be used to supplement my insurance	15 (79%)
General pediatric care as it relates to my child with CP (growth, sleep, nutrition, etc.)	15 (79%)
Knowledge surrounding pain in CP and how to handle it	15 (79%)
Roadmaps and reminder lists to help navigate all aspects of care during childhood for parents of children with CP	14 (74%)
Help telling what care is worth it and what is not	13 (68%)
Mental/behavioral health supports to help my child progress	11 (58%)
Preventive care to improve adult health	11 (58%)

#### 4.1.1 Importance of items related to child’s medical care

Ratings of select medical care options indicate that caregivers perceive as very important options such as in-home therapy and/or care services, easy access to CP-specialized medical providers, information about treatment/intervention options and their child’s developmental trajectory, and care coordinator support to help navigate service options (all Mdn = 100; 10th percentile values ranged from 50 to 85, all 90th percentile values = 100). At the same time, they rated telehealth options as only somewhat important (Mdn = 71, IPR 0–100).

#### 4.1.2 Difficulty accessing medical care

Caregivers rated medical appointments with primary care providers as somewhat easy (Mdn = 27, IPR 0–100). In contrast, “appointments with specialists” was rated somewhat hard (Mdn = 70, IPR 28–100). Access to other medical care needs, such as medications, equipment, and therapy were all rated as hard (Mdns = 50, IPRs 0–100).

#### 4.1.3 Preferences for therapy services

To identify caregivers’ preferred options for therapy services, we asked them to rate each one as if distance, availability, and cost were not barriers. Caregiver primary preferences were for individualized in-home options, followed by in-clinic options and a group setting that included children with similar issues (Table 3). The two options that were least preferred were telehealth options, as both weekly and intensive options were rated as somewhat not preferred (Mdn = 50, IPR 0–66 and Mdn = 59, IPR 0–92, respectively).

#### 4.1.4 Community opportunities

When asked to rate the importance of community-based experiences, caregivers rated as very important opportunities for social connection (Table 3). Caregivers deemed both staying informed about the latest CP care and enabling their child to connect with peers as extremely important (Mdn = 100; IPRs 50–100 for each item).

#### 4.1.5 Learning priorities

When caregivers rated their interest in learning about a range of CP-related topics (Table 4), almost all (95%) of caregivers endorsed wanting to know more about motor-focused interventions. More than 80% of caregivers valued learning more about environmental supports to aid in development, latest scientific developments relevant to CP care, and CP-specific family and social supports.

### 4.2 Clinician ratings

Clinician ratings of the importance of clinical objectives for establishing and maintaining high-quality CP clinical programs and the extent of their implementation are summarized in Table 2, along with clinician perceptions of barriers to clinical objective implementation at their institution and patient access to care more broadly. Georgia HRIF clinicians and EDI clinicians did not differ in ratings of importance, implementation, clinical barriers, or patient/family barriers to care on independent samples median tests (all *p*s >0.05), and so responses are combined in the present analysis.

#### 4.2.1 Importance of clinical objectives

There was strong agreement among clinicians that clinical objectives, such as connecting families with intervention services, family-centered approaches to care, addressing socio-economic barriers to family participation in care, and access to interdisciplinary medical teams are important for high-quality CP clinical programs (all Mdns = 100, IPRs 72–100).

#### 4.2.2 Degree of implementation in practice

At the same time, the degree to which clinicians perceive that these objectives are implemented was variable (Mdns = 56–90, IPRs = variable). Of the clinical objectives, “addressing psychological concerns for families” was the less consistently implemented (Mdn = 56, IPR 21–91) followed by “access to support services across the lifespan” (Mdn = 60, IPR 20–97) and efforts to address socio-economic resources issues for families” (Mdn = 62, IPR 31–94). At the same time, clinical objectives that were being more consistently implemented included patient-centered approaches to care (Mdn = 88, IPR 58–100), connecting families to intervention services (Mdn = 90, IPR 50–100), and use of evidence-based care practices (Mdn = 82, IPR 50–100).

#### 4.2.3 Barriers to implementation of clinical objectives

Ratings indicated that clinicians perceived the lack of the following to be “somewhat” barriers: clinician time (Mdn = 77, IPR 50–100), insurance reimbursement (Mdn = 75, IPR 25–100), readily identifiable resources for families (Mdn = 65, IPR 25–89), opportunities to learn evidence-based research (Mdn = 61, IPR 19–92), and established systematic approaches to care (Mdn = 61, IPR 15–100).

### 4.3 Caregiver focus group responses

As summarized in Table 5, thematic analysis of caregiver focus group responses identified three key themes related to obtaining medical care for their child: access to care needed, quality of care received, and feelings about their efficacy at obtaining care. Each theme, and associated subthemes, is described subsequently followed by discussion of the convergence between caregiver and clinician perceptions of patient barriers to CP clinical care (see Supplementary Table S3 for full definitions and additional quotes).

**Table 5:** Caregiver reported barriers faced when trying to get child high quality medical care, therapy services, or equipment: number (percent).

Theme/subtheme	# (%)^a^
Access to Appropriate Care	74 (51%)
Resource gap	23 (31%)
Dissatisfaction with available service options	15 (20%)
Insurance/Cost	13 (18%)
Long wait time	13 (18%)
Distance/Location	10 (13%)
Parent Disempowerment	40 (28%)
Bidirectional communication between caregiver and provider	15 (37%)
Centralized guidance/coaching for care of child	13 (33%)
Support for parent needs	12 (30%)
Customer Service Issue	30 (21%)
Process/System issue	23 (77%)
Quality of care	3 (10%)
Communication among specialty providers	2 (7%)
Perceived lack of care for child	2 (7%)

^a^Theme % out of total responses for question; Subtheme % out of theme total.

#### 4.3.1 Theme 1: access to care

Access-related responses described the ability of a patient/family to enter the clinical system (e.g., see a qualified health provider within a reasonable time). Caregiver responses identified logistical factors, such as distance to provider, cost, and wait time as barriers to existing clinical care options. At the same time, they also reported gaps in resources (e.g., “finding nursing care is impossible” and “hard to find primary care physician who could tend to child’s needs with multiple concerns”).

#### 4.3.2 Theme 2: quality of care

Quality*-*related responses described difficulty or dissatisfaction with the provision of services once in the system due to a lack of assistance or advice from a provider or patient-facing organization (e.g., clinic, hospital, government program). Within this theme, caregiver responses reflected dissatisfaction with available service options and organizational barriers within the care process (e.g., “getting providers to respond for care/schedule appointment w/o being redirected” and “identification of appropriate equipment but no response – ‘battle’ to actually obtain equipment”). Caregivers also reported challenges related to perceived quality of care received [e.g., “some specialists quick to diagnose w/o assessing baby’s state (hungry/tired)”], perceived lack of care for child (e.g., “[we experienced] delay in getting necessary equipment, [it was] not a priority to people in charge.”), and inter-specialty communication (e.g., “disconnect b/w developmental therapist and some programs, streamlining could be better”).

#### 4.3.3 Theme 3: feelings about care

The third theme reflected in caregiver responses described *feelings about care* and available services. Reponses within this theme reflected barriers preventing patient/families from feeling or being successful in the care of their child due to real or perceived lack of power, authority, or influence over the care priorities and plan for their child. These feelings of disempowerment were reflected in caregiver-identified needs for centralized coaching/guidance through critical transitions and recognition of social, emotional, and logistical factors faced by caregivers. Caregivers also reported the absence of or limited partnering with providers around care priorities (i.e., bidirectional communication ensuring parent/family perspectives are understood; “more respect [needed] for questioning parents, [parents who question are] labeled ‘non-compliant’ when seeking other options.” and “docs telling me things I already know.”).

#### 4.3.4 Clinician perceptions of barriers to patients and families

Clinicians were asked to rate barriers to patient/families engaging in clinical care (Table 6). The top barriers identified were social determinant of health-related (Mdn = 81, IPR 50–10); awareness (Mdn = 76, IPR 49–100) of and access (Mdn = 50–100) to clinic/clinician services; insurance coverage of services (Mdn = 75, IPR 20–100); and lack of knowledge of CP by caregivers (Mdn = 70, IPR 50–99). Perceived barriers also included lack of trust in the medical system (Mdn = 60, IPR 30–96), health literacy (Mdn = 70, IPR 39–95), and limited understanding of importance of clinical care/services (Mdn = 68.5, IPR 35–90).

**Table 6:** Clinician ratings of barriers to engaging patients with CP/families in clinical care: Scale: 1–100 with 100 being very much so: median (10th, 90th percentile).

Barrier	Median (10th, 90th)
Socio-economic status-related issues (e.g., lack of transportation, housing, childcare)	81 (50, 100)
Limited or no physical access to clinics or clinicians where they live	76 (49, 100)
Lack of awareness of clinical services	75 (50, 100)
Limited or no insurance coverage of clinical services	75 (25, 100)
Lack of knowledge surrounding their child’s condition	70 (50, 99)
Lack of trust in the medical system	60 (30, 96)
Health literacy or general literacy	70 (39, 95)
Limited family understanding of the importance of clinical care/services	68.5 (35, 90)
Lack of availability of these resources for the families’ needs (e.g., lack of space or appointments)	70 (38, 100)

Note: *N* = 102.

## 5 Discussion

This study sought to employ PAR methods to build and sustain patient-centered agendas for clinical care in CP (17). We report the perspectives of caregivers of a young child with CP and clinicians regarding CP-specific medical care priorities, and barriers and facilitators to high quality CP-focused medical care in Georgia, US.

### 5.1 Synergy and divergence

**Figure 2:**
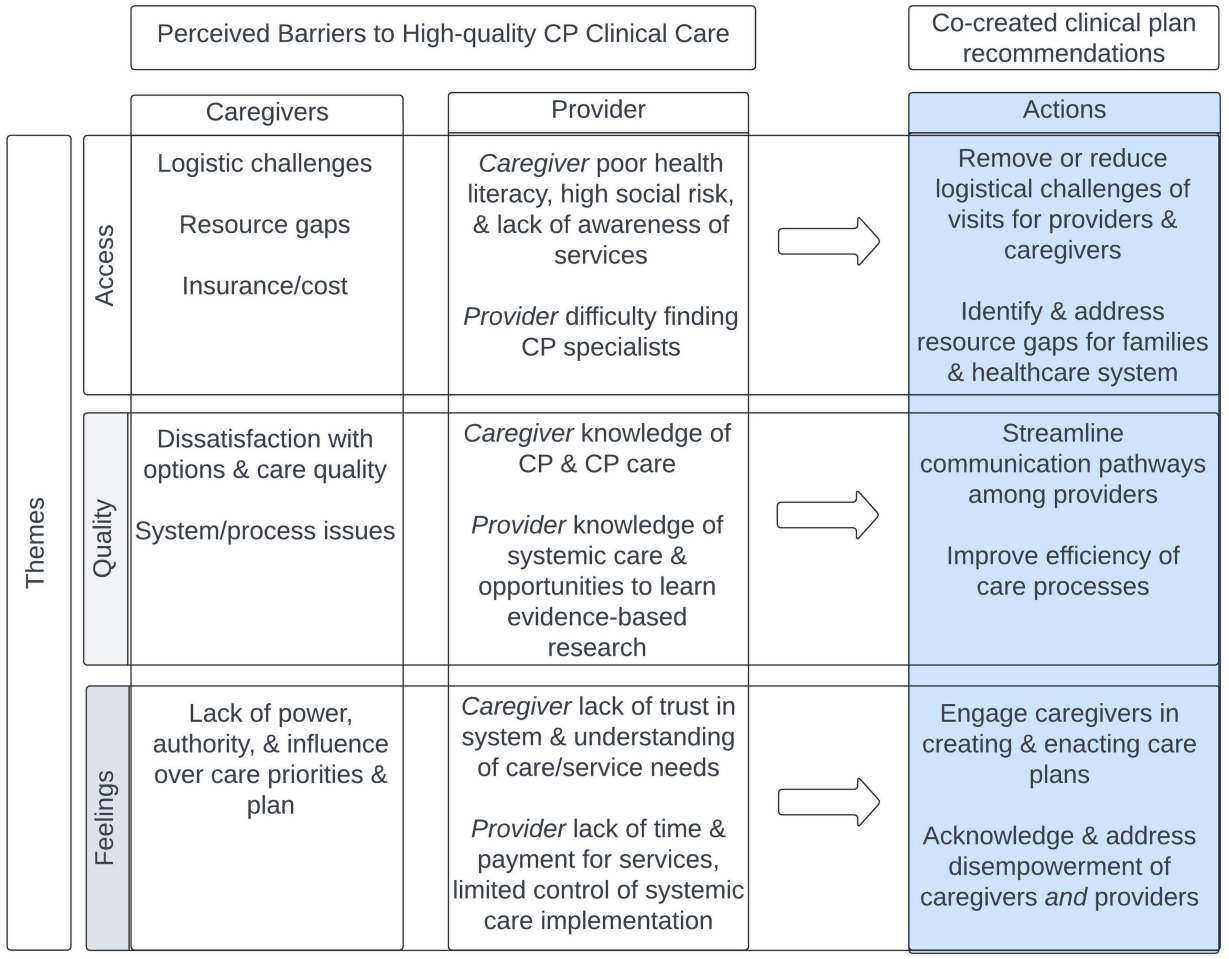
Stakeholder perceived barriers to CP clinical care by theme and co-created clinical plan recommended actions. The feelings of caregivers and providers tell us why change is needed. Quality of care tells us how the system should operate. The access barriers tell us what needs to change.

Both clinician and caregivers recognized that access barriers exist to high-quality CP care and contribute to perceived caregiver *and* provider feelings of disempowerment. However, both groups had different ideas about (1) what constitutes barriers to access and (2) the sources of the feelings associated with disempowerment (Figure 2). For their part, caregivers reported *logistical barriers* to clinical access. However, caregivers did not report that knowledge of CP, health literacy, or socio-economic issues were barriers – in contrast to clinicians who rated these as barriers for families. Similarly, caregiver descriptions of barriers related to disempowerment and encompassed feelings of powerlessness, dissatisfaction with care quality, and ineffectiveness at influencing care plans and priorities. Clinicians endorsed disempowerment-related barriers for caregivers but attributed different etiologies to them (e.g., lack of trust, understanding of care the child needed). However, the disempowerment clinicians acknowledged for their patients and families was mirrored by the ones they themselves felt, as expressed by their feelings of limited control over system-level care approaches, lack of time and lack of payment to provide services.

These apparent divergences highlight the importance of considering multiple stakeholder perspectives: the recurrent theme of disempowerment underscores *why* change is needed, the theme of quality describes *how* the system should operate, and the theme of access describes *what* elements of change are needed to build an empowered CP patient and provider community. As such, we can derive actionable priorities that can serve as a road map for improving the quality of care in CP clinical programs, and ultimately CP outcomes across the lifespan.

### 5.2 Proposed actions for systems change

Caregivers’ perceptions about barriers to obtaining high-quality CP care in the present study are consistent with recent reports from caregivers of a child with CP in Australia (12). Interviews with caregivers who experienced delayed access to early intervention (referral of child after 6 months adjusted age), identified themes that incorporated “insufficient communication and support” leading to disempowerment and uncertain trust, as well as “systemic barriers” leading to delays in access to care (12). Although these findings describe CP early detection-focused experiences of caregivers, barriers align with the feelings of disempowerment, perceived quality of care, and access-related challenges we identified when accessing healthcare more broadly, suggesting that removing barriers to early diagnosis alone is not sufficient to improve ongoing access to care needs for families.

Indeed, perceived barriers by caregivers must be tempered *by the reality* that there are not enough CP providers or resources at the local, state, or national levels – at least in the US. Therefore, an immediate solution is to *empower caregivers* to better utilize the resources that do exist and *empower providers* in efficient delivery of high-quality CP care.

#### 5.2.1 Empower caregivers

Consistent with other studies of parent perceptions of CP care, our participants reported the value and importance of caregiver-providers collaboration in establishing care plans and decision making (32, 33). Caregiver empowerment, as both a process and outcome, was also positively associated with child cognitive and motor outcomes for children under 3 years with CP (34). Combined with clinician support, it can also result in effective parent-delivered interventions across childhood (32, 35). In Table 7, we propose potential logistical and psychosocial approaches to addressing barriers and accelerating caregiver empowerment.

**Table 7:** Approaches to eliminating caregiver and provider endorsed barriers to high-quality CP care.

Group	Logistical approaches	Psychosocial approaches	Examples of implementing in action for Georgia, US
Caregiver	Traveling clinics to extend organizational service areas Leveraging primary care appointment availability to alleviate delays for specialist appointments Offering non-standard business hour appointments for working parents providing childcare support for during clinic visits for siblings	Incorporating care navigation support Creating processes for screening and addressing for caregiver mental health and other needs (e.g., social risk factors)	Partnership with Department of Health to conduct monthly HRIF surveillance by specialists into rural health department facilities Systematic screening for ACES, PTSD, social needs in all Atlanta region HRIF (42)
Provider	Improve efficiency of care processes Enhancing organizational processes to improve customer service Streamlining communication among providers Advocate for reimbursement for complex medical care diagnoses Co-created learning curricula available throughout clinician training pipelines Provision of evidence-based and implementable guidelines	Provide opportunities to learn/reinforce evidence-based research practice Easily available support for providers with fewer opportunities to learn CP care by experienced providers and specialists Consistent training in shared decision-making practices	Implementation of early detection guidelines for CP in all Atlanta Region HRIF Training of all other GA HRIF in early detection and intervention guidelines and QI initiatives supported by the Department of Health State-sponsored Scholarships to annual CP Early Health Summit skills workshops in Atlanta for state early intervention providers, physicians and trainees

Note: HRIF, High Risk Infant Follow up; ACEs, Adverse Childhood Experiences; CP, cerebral palsy; GA, Georgia US; PTSD, Post-traumatic Stress Disorder; QI, quality improvement.

#### 5.2.2 Build provider capacity and confidence

Responses from provider participants were consistent with those reported by others (36) and highlight the complexity of implementing and sustaining high-quality CP care. The importance providers placed on clinical objectives aligns with the 2022 report from the American Academy of Pediatrics (AAP) and American Academy for Cerebral Palsy and Developmental Medicine (AACPDM) guidelines and resources for strengthening access high-quality CP care (37). Among key guidelines were to educate and support pediatricians in early identification of CP as well as “increasing communication and collaboration with the child’s resource providers” and “educating caregivers” (p. 6). However, provider responses in the current study indicated opportunities to strengthen and accelerate implementation of these guidelines and clinical objectives in CP programs (Table 7). By deriving logistical and psychosocial approaches to addressing convergent themes, it is possible to derive an action plan for systems change, and test whether it is feasible at a state level. Examples of systems change in partnership with the state include Department of Health in Georgia and various healthcare organizations in the community (e.q., Early intervention providers, medical and allied health institutions).

**Figure 3:**
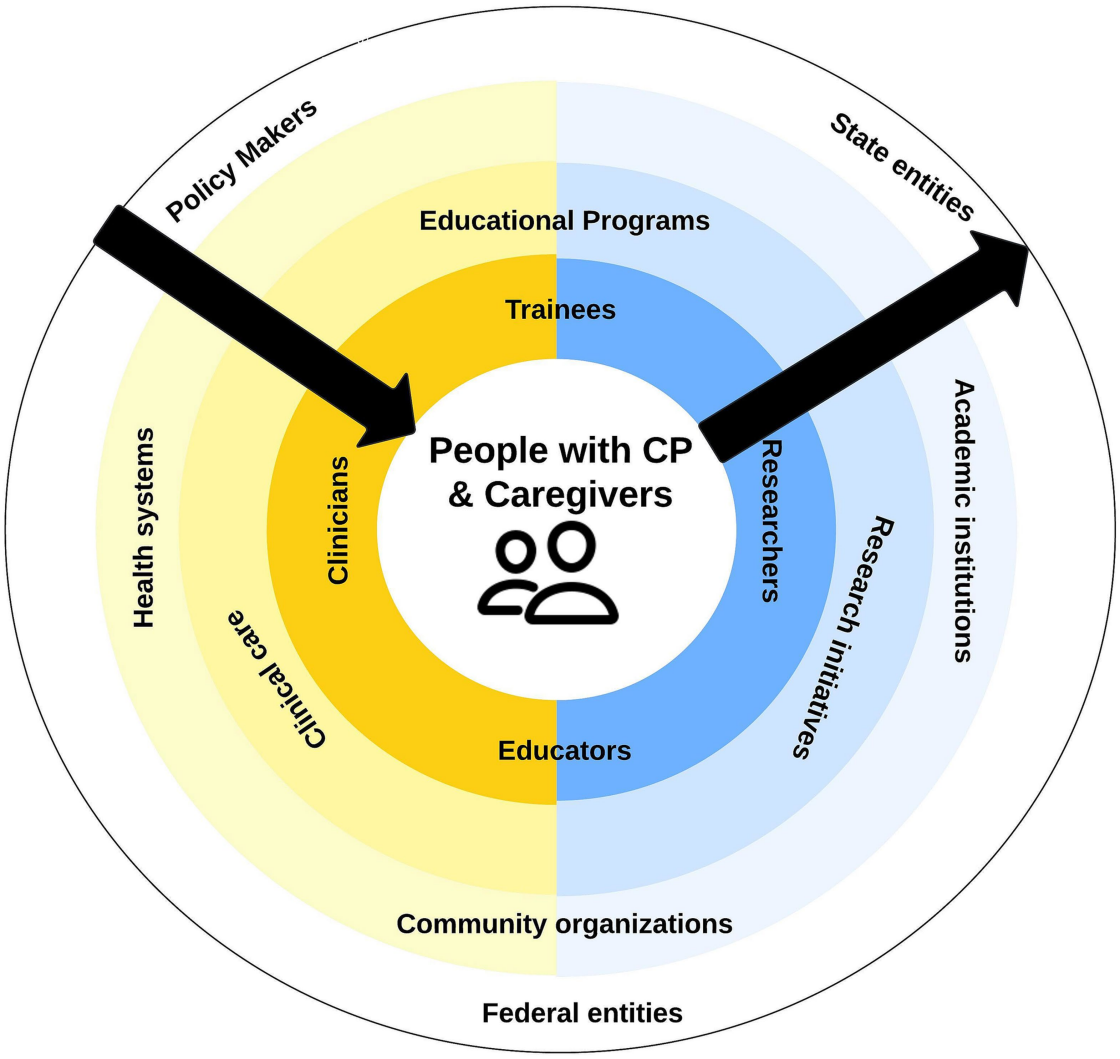
Directional progress required for system-level change in Figure 1.

The dual strategy of empowering caregivers alongside building provider capacity and confidence could move the entire system forward. Addressing access barriers, including those identified in this study, begins with caregivers and patients at the center of framework and works outward to drive change (Figure 3). It leads to the promise of increased caregiver capacity reserve, which can then be directed toward promoting improved individual-level outcomes and the potential for advocacy. Indeed, patient and parent-led foundations have been at the heart of early identification, intervention, research, and advocacy initiatives for neurodevelopmental disabilities such as fragile X syndrome (38, 39) and autism spectrum disorder (40, 41). And yet, CP is behind despite being most common lifetime physical disability.

At policy and health systems levels, alleviating provider perceived barriers to clinic implementation high-quality CP services and enhancing provider empowerment to provide interdisciplinary, evidence-based care that addresses patient/family needs begins to work from the outside of the system inward. Obtaining metrics and outcomes that matter at all levels of the ecosystem will also ensure progress toward the goal of improving the health and wellbeing of people with CP across the lifespan.

### 5.3 Limitations and next steps

The present initiative deployed mixed-method approaches with the goal of incorporating the strengths of both quantitative and qualitative research while offsetting limitations. PAR approaches with caregivers allowed for deep exploration of a sensitive topic but necessitated smaller sample sizes, which limits generalizability of study findings. For example, caregivers in the present initiative had children in the early years, and so themes associated with longer-term consequences of disempowerment and trauma (e.g., fear, powerlessness, and incapacitation) may have yet to emerge. Additional studies are needed to engage broader stakeholder bases to ensure the representativeness of findings in Georgia and inform progress nationally.

In contrast, surveys were used with providers to reduce burden and quickly reach a broader audience. However, even including open-ended questions, surveys lack the degree of personalization and in-depth exploration possible through focus groups. Regardless, the degree of convergence in themes between the groups remains striking, suggesting that an ability to triangulate responses from different sources and approaches can still be considered a strength of the present initiative. Additional research efforts will continue to evaluate this convergence across a broader range of stakeholders across the US.

## 6 Conclusion

Areas of synergy and discrepancy between the two stakeholder groups converge on the notion that (1) *empowering caregivers* to better utilize the resources that do exist and (2) *empowering providers* in efficient delivery of high-quality CP care are critical for improving access to care for people with CP. Efforts to facilitate lasting system-level changes may be accelerated through simultaneous addressing the needs of both groups. Increasing caregiver capacity by reducing burden has the potential to accelerate self- and community-advocacy for care access and clinical research that informs care. Similarly, building provider capacity can drive system-level changes that ultimately benefit all stakeholders and improve outcomes for the CP community across the lifespan.

## Data Availability

The raw data supporting the conclusions of this article will be made available by the authors, without undue reservation upon reasonable request.
