# Supplementary material for: Caregiver and clinician perceptions of barriers to cerebral palsy healthcare—mixed methods findings and systems change recommendations
Source: Front Public Health. 2025 Oct 22;13:1644144. doi: 10.3389/fpubh.2025.1644144 (PMC12586065; doi:10.3389/fpubh.2025.1644144)
Supplement: Supplementary file 1 [file Table_1.docx]

Supplemental Table S1 Caregiver survey

**Part I. Please answer the following questions about your child’s medical care.**

1. How important are the following items to you with regard to your child's medical care?

*Slider scale: 0 (not at all) to 100 (very much so)*

a. Easy access to medical providers that know about cerebral palsy

b. Easy access to the latest information about cerebral palsy and how it can affect my child's development

c. Easy access to the latest information about interventions and treatments for cerebral palsy

d. Options for in-home therapy and/or care services for my child

e. Options for telehealth services for my child

f. Understanding my child's options to care

g. A care coordinator to help me navigate all of these services

1. How hard is it for you to access the following items with regards to your child's medical care?

*Slider scale: 0 (not at all) to 100 (very much so)*

a. Occupational or Physical Therapy

b. Speech or Feeding Therapy

c. Medical appointment with primary care

d. Medical appointments with specialists (e.g., neurologists, developmental pediatricians)

e. Affordable medications

f. Therapeutic equipment (e.g., splints)

1. Is there anything else about your child's care that you would like to include?

**Part II Please answer the following about potential opportunities for your child and/or your family.**

1. How important are the following community opportunities to you?

*Slider scale: 0 (not at all) to 100 (very much so)*

a. For me (the caregiver) to have in-person opportunities to get to know other parents that have children with cerebral palsy

b. For me (the caregiver) to have opportunities online to get to talk to other parents that have children with cerebral palsy

c. For my child to have opportunities to get to know other children with cerebral palsy

d. For my family to have opportunities to learn more about the latest updates in caring for a child or person with cerebral palsy

e. For my family to have opportunities to participate in studies that help us learn more about cerebral palsy

1. Generally, which (if any) of the following topics related to cerebral palsy would you want to learn more about? [select all that apply]

- General pediatric care as it relates to my child with CP (growth, sleep, nutrition, etc.)
- Preventive care to improve adult health
- Environmental supports to help my child progress
- Community/state supports to help my child progress
- Extra resource options that can be used to supplement my insurance
- Mental/behavioral health supports to help my child progress
- Family and social supports specialized to those with children with CP
- Roadmaps and reminder lists to help navigate all aspects of care during childhood for parents of children with CP
- Interventions to improve motor function (e.g., therapy, surgery, medicine)
- Knowledge surrounding pain in CP and how to handle it
- News updates when something related to my child's CP happens
- Help telling what care is worth it and what is not

**Part III Please answer the following question about how Early Intervention services could be available to you and your child.**

1. When thinking about how you would want early intervention services provided to you, if the distance or availability of provider were not an issue, and there were no extra costs - how much would you prefer the following types of therapy services:

*Slider scale: 0 (not at all) to 100 (very much so)*

a. In the home – on a weekly basis

b. In the home - intensive periods for a few weeks followed by rest periods to consolidate skills

c. In a group setting with families who have children with the same issues

d. In the clinic - on a weekly basis

e. In the clinic - intensive periods for a few weeks followed by rest periods to consolidate skills

f. Via telehealth - on a weekly basis

g. Via telehealth - intensive periods for a few weeks followed by rest periods to consolidate skills

h. Add your own method of service delivery ______

1. Is there anything else about your family's engagement with the CP Community that you would like to include?

Supplemental Table S2. Clinicians

Clinical Practice Questions

When answering the following questions: Think back over your experience as a clinician treating patients with CP over the last 5 years or so….

*Slider scale: 0 (not at all) to 100 (very much so)*

1. How important do you feel the following clinical objectives are to creating and maintaining a high quality program in treating patients with CP?

a. Interdisciplinary approach (e.g., medical doctors/nurse practitioners work together with therapists and other specialists to provide and coordinate care)

b. Use of evidence-based care and practices

c. Using a patient/family-centered approach to care

d. Early detection and diagnosis for patients with cerebral palsy

e. Connecting families with appropriate intervention services (e.g., early intervention, Babies Can't Wait, PT/OT/SLP referrals, specialist referrals)

f. Providing access to support services across the lifespan for people with cerebral palsy

g. Addressing socio-economic resource issues for families and patients with cerebral palsy

h. Addressing psychological concerns for families and patients with cerebral palsy

i. Add your own clinical objective

2. How frequently does your clinical program implement the following 'clinical objectives' in its efforts to establish, maintain, or contribute to a high-quality clinical program in treating patients with CP at your institution?

a. Interdisciplinary approach (e.g., medical doctors/nurse practitioners work together with therapists and other specialties to provide and coordinate care)

b. Use of evidence-based care and practices

c. Using a patient/family-centered approach to care

d. Early detection and diagnosis for patients with cerebral palsy

e. Connecting families with appropriate intervention services (e.g., early intervention, Babies Can't Wait, PT/OT/SLP referrals, specialist referrals)

f. Providing access to support services across the lifespan for people with cerebral palsy

g. Addressing socio-economic resource issues for families and patients with cerebral palsy

h. Addressing psychological concerns for families and patients with cerebral palsy

i. [Clinical objective indicated in 1i]

3. How much do you think the following are or would be barriers to implementing these 'clinical objectives'?

a. Lack of clinician time

b. Lack of understanding and opportunities to learn evidence-based research and practice

c. Lack of insurance reimbursement and payment for services

d. Not a good systematic approach to care in place

e. Families not engaged in attending appointments

f. Hard to find or access most up-to-date research

g. Hard to find or access the correct specialists

h. Hard to find appropriate resources for the family

i. Add your own barrier

4. How much do you think the following areas are or would be barriers to patients with CP/families engaging in clinical care?

a. Limited or no insurance coverage of clinical services

b. Limited or no physical access to clinics or clinicians where they live

c. Lack of awareness of clinical services

d. Socio-economic status-related issues (e.g., lack of transportation, housing, child care)

e. Lack of trust in the medical system

f. Lack of knowledge surrounding their child's condition

g. Health literacy or general literacy

h. Limited family understanding of the importance of clinical care/services

i. Lack of availability of these resources for the families' needs (e.g., lack of space or appointments)

j. Add your own barrier

5. Is there anything else you would like to add about what is working or could be improved related to your experience in clinical practice?

| *Supplemental Table S3. Questions 1 & 2 Main theme and sub-theme frequency, definitions, and examples from Caregiver World Café discussions* | | | |
| --- | --- | --- | --- |
| **Theme/Subtheme** | **Definition** | **Example** |  |
| **Access to Appropriate Care** | Disruption in the ability of a patient/family to see a qualified health provider within a reasonable time period. | |  |
| Distance/Location | A resource exists, but family perceives it is too far away to be reasonably accessible. | *No ED [emergency department] or specialists south of downtown Atlanta  Distance to appointments* |  |
| Insurance/Cost | Problems with insurance coverage and/or high out-of-pocket costs for resource that dictate access to service or continuity of service/provider. | *Insurance is a hurdle to get scheduled appointments* |  |
| Long Wait Time | A resource exists, but the wait time to receive resource is unreasonably long (i.e., excessively delays care). | *appointment cancellations cause huge time gaps between visits - provider availability  It takes forever to get appointments... sometimes 6 months* |  |
| Resource Gap | A resource doesn’t exist or isn't available at all to the family. If a resource does exist, it cannot be reliably accessed or accessed at a sufficient dose to be effective. The access difficulty is not due to distance, insurance, or wait time. | *No consistent therapy.  lack of availability of durable medical devices and equipment   One family was told by [local agency] to "I advise you to stay with [regional provider] because they [local agency] don't have providers"* |  |
| Dissatisfaction with available service options | A service can be accessed but does not meet caregiver's perceived want/need. | *[Transferring care causes] no continuity of care [and prevents] building a relationship with the therapist, not just patients but parents too*   *"Telehealth was challenging with young children...There are also issues with interference, "distractions in the home ... such as other kids and pets."* |  |
| **Customer Service Issue** | Difficulty accessing care due to a lack of assistance or advice from a provider or patient-facing service organization (e.g., clinic, hospital, government program). Response must focus on the process | |  |
| Communication among specialty providers | Lack of clear and consistent communication among different providers and/or organizations. | *Parent has to get physicians to collaborate with each other. [patient portal] helps but even so [and doesn't find patient portal to be as effective]* |  |
| Perceived lack of care for child | Provider and/or organization is perceived by caregiver as uncaring for child/family needs in the context of a visit or service encounter. | *Delay in getting necessary equipment, not a priority to people in charge* |  |
| Process/system issues | Organizational barriers to care including disorganized or inefficient processes (e.g., repeating medical history, resource coordination) and poor service integration among specialties. Response must focus on the process or people that are providing/coordinating the care. | *Schedulers are not reaching out to families (specifically Specialty Areas 1, 2, and 3)* |  |
| Quality of care | Poor medical care delivery perceived by caregiver | *Had to go into medically induced coma because they did not change feeding tube: hospital negligence cause a lot of bigger problems* |  |
| **Parent Disempowerment** | Barriers preventing or hindering patient/families from feeling or being successful in the care of their child due to real or perceived lack of power, authority, or influence over the care priorities and plan for their child. | |  |
| Bidirectional communication between caregiver and provider | Absence of or limited partnering with patients/families in all stages of care to set care priorities; ensuring patient/family perspectives are understood; Response must include intentional emphasis on collaboration (or lack of collaboration) rather than imparting information or having to fight with providers for care or information. | *“We parents are researching more than doctor”  They never get an option, they feel like they're being forced to go a certain medical route.* |  |
| Centralized guidance/coaching for care of child | A need for centralized guidance/coaching through critical transitions (e.g., NICU to home) and overall care journey (e.g., Roadmap to care). | *having a case manager to help caregivers navigate options and make care plan.* |  |
|  |  |  |  |
| Support for parent needs | A need to recognize and address caregiver social, emotional, and logistical needs (e.g., balancing work and home demands, caring for siblings). Emphasis may be on caregiver emotional state, mental health, or well-being. | *For busy parents/families, need support navigating system/studies/ intervention.* |  |

1. Murphy MM, Colquitt GT, Ryals PS, et al. Synergies, Discrepancies, and Action Priorities: A Statewide Engagement Study to Strengthen Clinical Research in Cerebral Palsy. *Health Expect*. Jun 2025;28(3):e70257. doi:10.1111/hex.70257

2. Kendrick-Allwood S, Murphy M, Maitre N, Walker L, Shin K. Social determinants of health on the intervention in early cerebral palsy. *Developmental Medicine & Child Neurology*. 2023;65(S3):185. doi:<https://doi.org/10.1111/dmcn.15702>
